# Pericyte Contractile Responses to Endothelin-1 and Aβ Peptides: Assessment by Electrical Impedance Assay

**DOI:** 10.3389/fncel.2021.723953

**Published:** 2021-08-20

**Authors:** Elliott Hibbs, Seth Love, J. Scott Miners

**Affiliations:** Dementia Research Group, Clinical Neurosciences, University of Bristol, Bristol, United Kingdom

**Keywords:** Aβ peptide, Alzheimer’s disease, electrical impedance, endothelin-1, pericyte

## Abstract

Pericytes are vascular mural cells that contract and relax in response to vasoactive stimuli to regulate neurovascular coupling and cerebral blood flow. Pericytes are damaged and degenerate in Alzheimer’s disease (AD). We previously showed that the level of the regulatory vasoconstrictor, endothelin-1 (EDN1), is elevated in AD cerebral cortex and upregulated by amyloid-beta (Aβ). We have used electrical impedance analysis to monitor the contractile and proliferative response of cultured human fetal and adult brain-derived pericytes to EDN1 in real-time. EDN1 caused transient, dose-dependent contraction of fetal and adult brain pericytes that was mediated by EDN1 type A receptors and increased the subsequent proliferation of fetal but not adult cells. The contractile responses to EDN1 were weaker in the adult pericytes. The EDN1-mediated contractile response of fetal pericytes was unchanged after exposure to Aβ_1__–__40_ or Aβ_1__–__42_ (0.1–10 μM) for 1 h but both contraction and subsequent relaxation were significantly impaired upon exposure to Aβ for 24 h. These data suggest that chronic exposure to Aβ interferes with EDN1-mediated pericyte contractility, potentially contributing to neurovascular uncoupling and reduced cerebral blood flow in AD.

## Introduction

Pericytes are vascular mural cells that are particularly abundant within the brain. They are expressed within the capillary network but also reside on pre- and post-capillary vessels. Pericytes are critical components of the neurovascular unit and dynamically regulate cerebral blood flow (CBF) in response to local metabolic demand (Joyce et al., 1985; Neuhaus et al., 2017; Nortley et al., 2019). In stroke, pericytes die in rigor, clamping shut capillary lumina, contributing to ischemic injury (Hall et al., 2014; Heyba et al., 2019). Recent studies indicate that pericytes are damaged and degenerate in Alzheimer’s disease (AD) and vascular dementia (VaD) (Sagare et al., 2013; Kisler et al., 2017; Ding et al., 2020). This is thought to play a major role in blood-brain barrier (BBB) breakdown and cerebral hypoperfusion, and to affect clearance of Aβ in AD (Daneman et al., 2010).

Brain ischemia has long been recognized as the principal pathological process in VaD but recent studies have shown that cerebral hypoperfusion and BBB leakiness are early and substantial contributors to disease progression and cognitive decline in AD (Ding et al., 2020). We previously showed that the enzymes responsible for synthesis of the vasoconstrictor peptide endothelin-1 (EDN1) were upregulated in AD (Palmer et al., 2009, 2013) and associated with elevated EDN1 level in the cerebral cortex. The level of EDN1 correlated strongly with biochemical evidence of cerebral hypoperfusion in AD and with the level of Aβ, which we found to increase the production of EDN1 by neurons and endothelial cells *in vitro* (Palmer et al., 2012). EDN1 induces contraction of smooth muscle cells, causing constriction of penetrating arteries and arterioles and reducing blood flow in the cerebral cortex and deep within the brain (Wynne et al., 2009). A recent study confirmed that capillaries were constricted in proximity to Aβ plaques in human biopsy brain tissue and that mechanistically, reactive oxygen species in response to oligomeric Aβ mediated EDN1-induced pericyte contraction and consequent narrowing of capillaries in brain cortical slices, and was likely to contribute to cerebral hypoperfusion in AD (Nortley et al., 2019).

*In vitro* studies of pericyte function have been performed with bovine retinal pericytes (Yamagishi et al., 1993) using semi-quantitative methods, such as the “wrinkling assay” [pericyte contraction causes folds when the cells are grown on a silicone membrane (Durham et al., 2014)], and with brain-derived human pericytes isolated from fetal brain tissue (*ScienCell, United States*) (Persidsky et al., 2016; Neuhaus et al., 2017). The fetal cells express pericyte markers such as platelet-derived growth factor receptor-β (PDGFRβ) and chondroitin sulfate proteoglycan-4 (NG2) and are negative for endothelial markers and non-vascular markers including GFAP and IBA1 (Hall et al., 2014). The cells respond to vasoactive metabolites and contract and migrate in culture (Neuhaus et al., 2017; Nortley et al., 2019; Kemp Scott et al., 2020). They have been shown to express α-smooth muscle actin (α-SMA) (Nehls and Drenckhahn, 1991).

Fetal pericytes are responsive to multiple vasoactive peptides in the brain, and contract in response to EDN1 as previously shown using an electrical impedance assay (Neuhaus et al., 2017). It remains unclear whether the pathophysiological responses of fetal brain pericytes reflect those in the adult brain. EDN1 also evokes pericyte-mediated constriction of capillaries in human and rat cortical slices in response to oligomeric Aβ (Nortley et al., 2019). In the present study, we have assessed EDN1-mediated contraction of fetal and adult brain-derived pericytes by use of an electrical impedance assay and investigated whether the contractile response is modified by exposure of pericytes to Aβ peptides.

## Materials and Methods

### Pericyte Culture

Human brain vascular pericytes of fetal origin (fHBVP; *ScienCell, United States*) and human brain vascular pericytes of adult origin (aHBVP; *Cell Systems, United States*) were grown in pericyte medium (*ScienCell, United States*) at 37°C in 5% CO_2_. All experiments were performed using cells at passages of <8. Cells were cultured in poly-L-lysine-coated flasks (10 mg/mL) until 80% confluent and then sub-cultured following the manufacturer’s protocol. All work was performed in a Class II microbiological safety cabinet (*Holten, United Kingdom*) under sterile conditions.

### Immunofluorescent Labeling of Pericytes

Immunofluorescence was used to assess the expression and distribution of cell-specific markers. Cells were cultured on poly-L-lysine coated coverslips and left to grow for 24 h at 37°C in a 5% CO_2_ incubator. The medium was aspirated, and cells were washed gently three times with PBS before being fixed in 4% paraformaldehyde (PFA; *Sigma-Aldrich, United States*) for 10 min at 37°C in a 5% CO_2_ incubator. PFA was removed and cells were washed three more times in PBS and left in PBS at 4°C until immunolabelled. At this point, PBS was removed, cold methanol added, and the cells incubated at −20°C for 10 min, washed with PBS 3 times for 1 min each and then blocked with 5% donkey serum (*Sigma-Aldrich, United States*) for 30 min at room temperature (RT). The cells were incubated with primary antibody overnight at 4°C: α-SMA (1:100, *Abcam United Kingdom, rabbit polyclonal*), PDGFRβ (1:500, *R&D Systems United States, biotinylated goat polyclonal*), EDNRA (1:500, *Abcam United Kingdom, rabbit polyclonal*), and EDNRB (1:1000, *Abcam United Kingdom, rabbit polyclonal*). The cells were washed again in PBS three times for 1 min each followed by addition of a donkey anti-rabbit IgG Alexa Fluor 488 (*Invitrogen, United Kingdom*) secondary antibody (1:500) for α-SMA and streptavidin Alexa Fluor 488 conjugate (*Invitrogen, United Kingdom*) for PDGFRβ for 1 h at RT in the dark. Samples were washed in PBS three times for 1 min each and coverslips were placed onto glass microscope slides and mounted using Vectashield mounting medium with DAPI (*Vector Labs, United States).* Slides were stored at 4°C in the dark before being imaged by confocal microscopy (*Nikon Eclipse 80i*).

### EDN1-Mediated Contraction of Pericytes Assessed Using an Electrical Impedance Assay (xCELLigence)

Pericytes (fHBVP and aHBVP) were seeded at 5000 cells/well onto pre-coated poly-L-lysine impedance plates (E-plate VIEW^®^ 96 or E Plate insert 16^®^, *ACEA Biosciences, United States*) and allowed to adhere for 1 h at RT within the hood and then overnight in the incubator at 37°C, 5% CO_2_. Cells were then starved in serum-free medium for 2 h. EDN1 (*Abcam, United Kingdom*) was added *in situ* and the cell index (see below) was measured using either the xCELLigence^®^ RTCA S16 or SP96-well plates (*ACEA Biosciences, United States*) maintained at 37°C in a 5% CO_2_ incubator. The cell index was recorded in real-time throughout the duration of the experiment. Addition of serum-free medium was used as a vehicle control. The relative impedance is presented as a “cell index” (an arbitrary value derived from the impedance software) which reflects the contact surface area between cells and the culture dish and the resistance to electrical current (see Supplementary Figure 1); the average normalized cell index for a particular well is generated by normalizing the raw cell index data with reference to the mean index across duplicate vehicle control wells. Upon addition of EDN1, parameters for the xCELLigence software were set to record in 5 s intervals for a total duration of 2 h (contraction-relaxation phase) and then 1 h intervals for a further 48 h (proliferation phase). Data analyses were performed using RTCA Data Analysis Software 1.0 (*ACEA Biosciences, United States*). The change in slope (1/h) of the cell index curve, to reflect the rate of responses, was calculated using the built-in function of the data analysis software. Measurements were recorded in duplicate wells for each condition; “*n*” refers to the number of independent experimental repeats.

To assess the effect of receptor blockers on EDN1-mediated contraction, BQ123 (*Tocris, United Kingdom*) and BQ788 (*Sigma-Aldrich, United States*) were added in serum-free medium, 1 h before the addition of EDN1.

To assess the effect of Aβ_1__–__40_ (*rPeptide, United States*) and Aβ_1__–__42_ (*rPeptide, United States*) on EDN1-mediated contraction, freshly solubilized Aβ peptides prepared in 35% acetonitrile and diluted in serum-free medium were added either 1 or 24 h prior to the addition of EDN1.

### Statistical Software

All statistical analyses were performed using GraphPad Prism 8.1.2 software package. Unpaired two-tailed *t*-tests or ANOVA with Dunnett’s multiple comparisons *post hoc* tests were selected and used for comparisons between groups as appropriate. *P*-values of <0.05 were considered statistically significant.

## Results

### Pericytes Isolated From Fetal and Adult Human Brain Express Pericyte Markers

**FIGURE 1:**
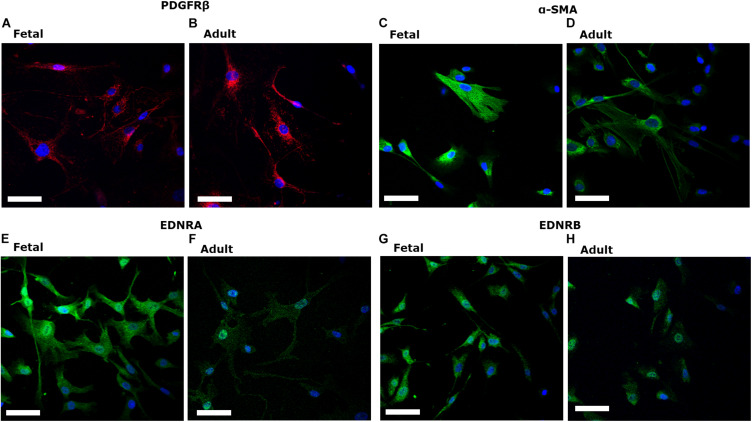
Fetal human brain-derived vascular pericytes (fHBVP) and adult human brain-derived vascular pericytes (aHBVP), expressed PDGFRβ **(A,B)**, α-SMA **(C,D)** and both endothelin-1 type A (EDNRA) **(E,F)** and type B (EDNRB) receptors **(G,H)**. All counterstained with a nuclear marker (DAPI – blue). Magnification 40×. Scale bar represents 50 μM.

fHBVP (*ScienCell*, *United States*) and aHBVP (*Cell-Systems, United States*) expressed the canonical pericyte marker PDGFRβ (Figures 1A,B). Pericytes expressed α-SMA (Figures 1C,D) and both endothelin-1 type A (EDNRA) (Figures 1E,F) and type B (EDNRB) receptors (Figures 1G,H).

### EDN1-Induced Contraction and Proliferation of Fetal Brain-Derived Pericytes

**FIGURE 2:**
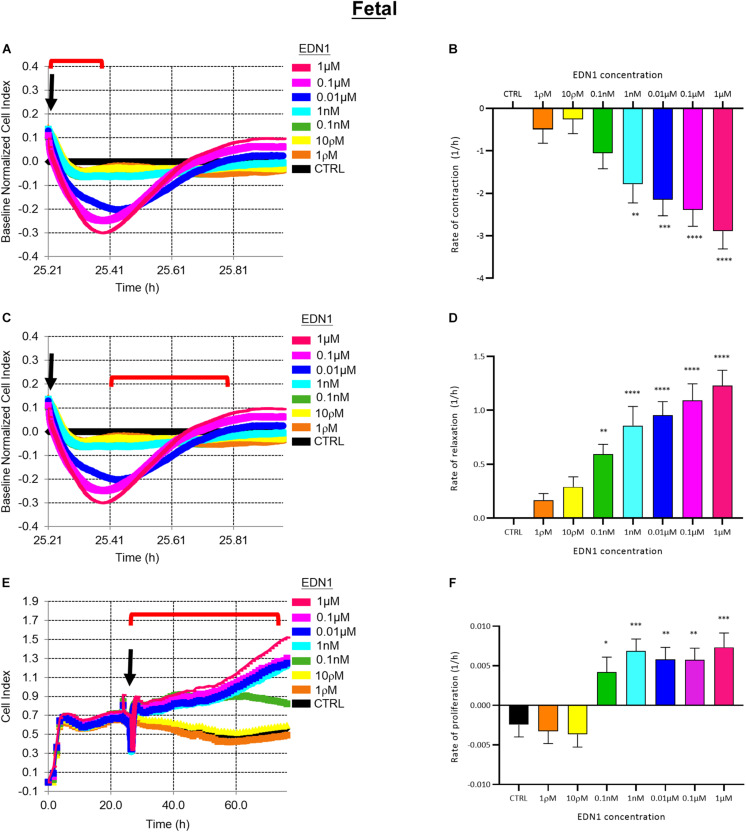
**(A)** Representative electrical impedance measurements of the contractile response of fetal human brain vascular pericytes (fHBVP) to EDN1. **(B)** The rate of contraction was significantly increased by addition of 1 nM (*p* = 0.0039, Dunnett’s test), 0.01 μM (*p* = 0.0003), 0.1 μM (*p* < 0.0001) or 1 μM (*p* < 0.0001) EDN1 (*n* = 12). **(C)** Representative electrical impedance measurements of the relaxation response of fHBVP following contraction. **(D)** The increased rate of contraction was followed by more rapid relaxation in cells treated with 0.1 nM (*p* = 0.0085), 1 nM (*p* < 0.0001), 0.01 μM (*p* < 0.0001), 0.1 μM (*p* < 0.0001) or 1 μM (*p* < 0.0001) (*n* = 12). **(E)** Representative electrical impedance measurements of the proliferative response of the fHBVP after EDN1. **(F)** Proliferation was significantly increased after the addition of 0.1 nM (*p* = 0.0290), 1 nM (*p* = 0.0008), 0.01 μM (*p* = 0.0038), 0.1 μM (*p* = 0.0041), or 1 μM (*p* = 0.0004) EDN1 (*n* = 12). The bars represent the mean values and SEM. Arrows indicate time of EDN1 addition. The timeframe referred to in each graph is indicated by the red bracket.

Electrical impedance assays (xCELLigence) were used to assess pericyte contraction, relaxation, and proliferation in response to EDN1, as previously reported (Neuhaus et al., 2017). Immediately after addition of EDN1, pericyte contraction caused a rapid decrease in cell index (relative impedance), followed by a relaxation phase (∼10 min) – a classic G-protein coupled receptor response – and subsequent proliferation, leading to a progressive rise in cell index over 48–72 h (Figure 2).

The rate of contraction of fHBVPs increased with the concentration of EDN1 (1 ρM–1 μM) as illustrated in Figure 2A. The initial rapid decrease in cell index over control values (cells in pericyte medium only) was significant after exposure to 1 nM (*p* = 0.0039), 0.01 μM (*p* = 0.0003), 0.1 μM (*p* < 0.0001), or 1 μM (*p* < 0.0001) EDN1 (*n* = 12) (Figure 2B). Contraction was followed by an increase in cell index (i.e., relaxation) as illustrated in Figure 2C. The rate of increase was related to the previous rate of decrease and was significantly higher in cells that had been exposed to 0.1 nM (*p* = 0.0085), 1 nM (*p* < 0.0001), 0.01 μM (*p* < 0.0001), 0.1 μM (*p* < 0.0001), or 1 μM (*p* < 0.0001) EDN1 (*n* = 12) (Figure 2D).

To monitor the effects of EDN1 on proliferation, the impedance was monitored for a further 48–72 h (Figure 2E), during which impedance rose reflecting an increase in the number of cells covering the plate. The rate of increase was calculated and indicated that proliferation of fHBVP was significantly greater than in untreated control cells when exposed to 0.1 nM (*p* = 0.0290), 1 nM (*p* = 0.0008), 0.01 μM (*p* = 0.0038), 0.1 μM (*p* = 0.0041), or 1 μM (*p* = 0.0004) EDN1 (*n* = 12) (Figure 2F). We similarly showed proliferation of fHBVPs in response to EDN1 using a standardized BRDU assay (Supplementary Figure 2).

**FIGURE 3:**
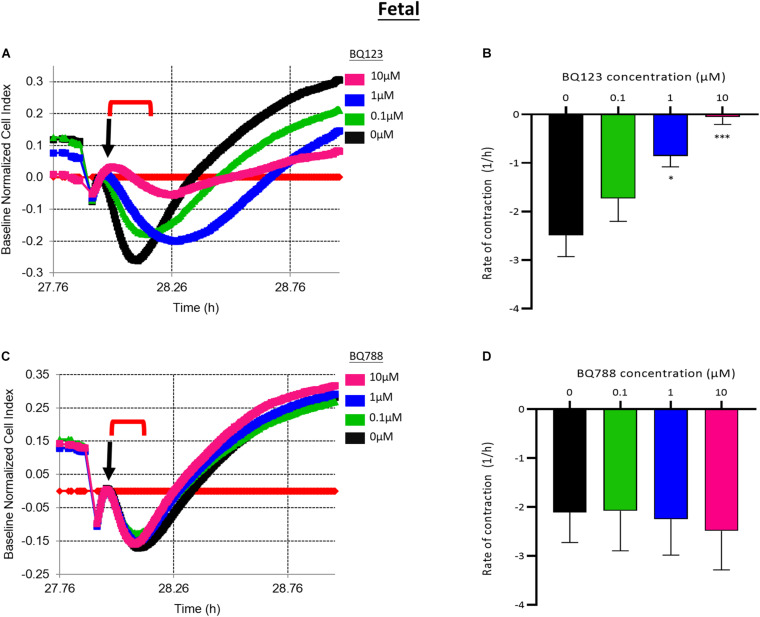
**(A)** Representative electrical impedance measurements of the contractile response of fetal human brain vascular pericytes (fHBVP) to EDN1 in the presence of BQ123, an EDNRA antagonist. **(B)** The rate of contraction was significantly reduced in cells to which 1 μM (*p* = 0.0158) or 10 μM (*p* = 0.0001) BQ123 had been added (*n* = 7). **(C)** Representative electrical impedance measurements of the contractile response of fHBVP in the presence of BQ788, an EDNRB antagonist. **(D)** There was no significant difference in the rate of contraction between BQ788-treated and untreated cells (*n* = 7). The timeframe referred to in each graph is indicated by the red bracket. Arrows indicate the timepoint of EDN1 addition. The bars represent the mean values and SEM.

Pretreatment with BQ123, an EDNRA antagonist, inhibited EDN1-mediated pericyte contraction in a dose-dependent manner (Figure 3A). A significant reduction in the rate of contraction induced by EDN1 (100 nM) was observed in cells pre-treated with BQ123 at 1 μM (*p* = 0.0158) or 10 μM (*p* = 0.0001) (*n* = 7) (Figure 3B). Pre-incubation with BQ788, an EDNRB receptor antagonist, did not significantly alter EDN1-induced pericyte contraction (*n* = 7) (Figures 3C,D).

### Adult Brain-Derived Pericytes Contract in Response to EDN1 via EDNRA but Do Not Proliferate

**FIGURE 4:**
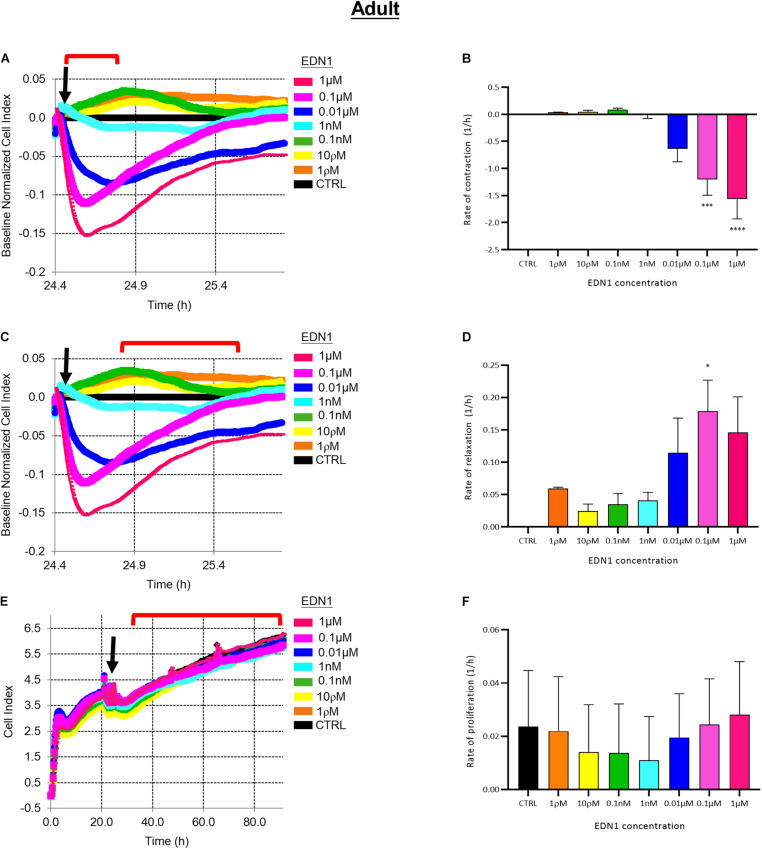
**(A)** Representative electrical impedance measurements of the contractile response of adult human brain vascular pericytes (aHBVP) to EDN1. **(B)** The rate of contraction was significantly higher after adding 0.1 μM (*p* = 0.0008) or 1 μM (*p* < 0.0001) EDN1 (*n* = 4). **(C)** Representative electrical impedance measurements of the relaxation response of aHBVP following contraction. **(D)** The rate of subsequent relaxation was significantly higher in cells to which 0.1 μM (*p* = 0.0159) EDN1 had been added (*n* = 4). **(E)** Representative electrical impedance measurements of the proliferative response of the aHBVP to EDN1. **(F)** No significant difference in rate of proliferation between EDN1-treated and control cells was found (*n* = 4). The timeframe referred to in each graph is indicated by the red bracket. Arrows indicate the timepoint of EDN1 addition. The bars represent the mean values and SEM.

EDN1 induced contraction of aHBVP. The rate of initial decline of cell index (i.e., contraction) was dose-related (1 ρM–1 μM), as illustrated in Figure 4A. The rate of contraction was significantly higher in adult cells treated with 0.1 μM (*p* = 0.0008) or 1 μM (*p* < 0.0001) EDN1 than in untreated cells (*n* = 4) (Figure 4B). The initial contraction was followed by a slower increase in cell index (i.e., relaxation), as shown in Figure 4C; the increase in cell index was most rapid after 0.1 μM EDN1 exposure (*p* = 0.0159) (*n* = 4) (Figure 4D).

Impedance was monitored for a further 48 h to assess the effect of EDN1 on adult pericyte proliferation. The cell index increased slowly (Figure 4E) and was not affected by exposure to EDN1 (*n* = 4) (Figure 4F).

**FIGURE 5:**
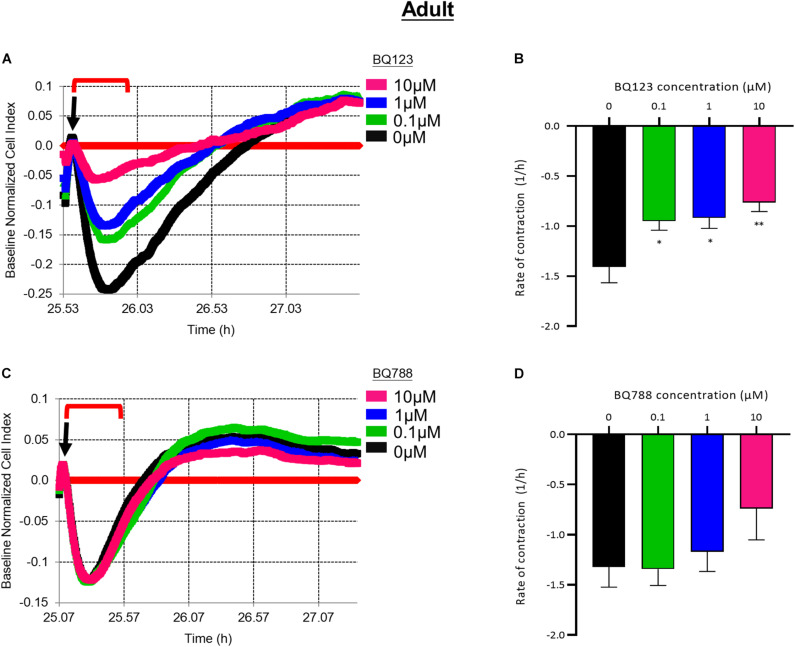
**(A)** Representative electrical impedance measurements of the contractile response of adult human brain vascular pericytes (aHBVP) to EDN1 in the presence of BQ123, an EDNRA antagonist. **(B)** The rate of contraction was significantly reduced in cells to which 0.1 μM (*p* = 0.0241), 1 μM (*p* = 0.0154), or 10 μM (*p* = 0.0016) BQ123 had been added (*n* = 7). **(C)** Representative electrical impedance measurements of the contractile response of aHBVP in the presence of BQ788, an EDNRB antagonist. **(D)** There was no significant difference in the rate of contraction between BQ788-treated and untreated cells (*n* = 7). Data shows mean rate of contraction ± SEM. The timeframe referred to in each graph is indicated by the red bracket. Arrows indicate the timepoint of EDN1 addition. The bars represent the mean values and SEM.

Pre-treatment with BQ123 inhibited EDN1-mediated contraction of aHBVP (Figure 5A). A significant reduction in the rate of contraction induced by EDN1 (100 nM) was seen in pericytes pre-treated with 0.1 μM (*p* = 0.0154), 1 μM (*p* = 0.0241), or 10 μM (*p* = 0.0016) BQ123 compared to cells treated with EDN1 alone (*n* = 7) (Figure 5B). Pre-treatment with BQ788 did not significantly influence EDN1-induced adult pericyte contraction (Figures 5C,D).

### EDN1-Induced Contractile Response of Fetal Pericytes Was Impaired by Exposure to Aβ Peptides

**FIGURE 6:**
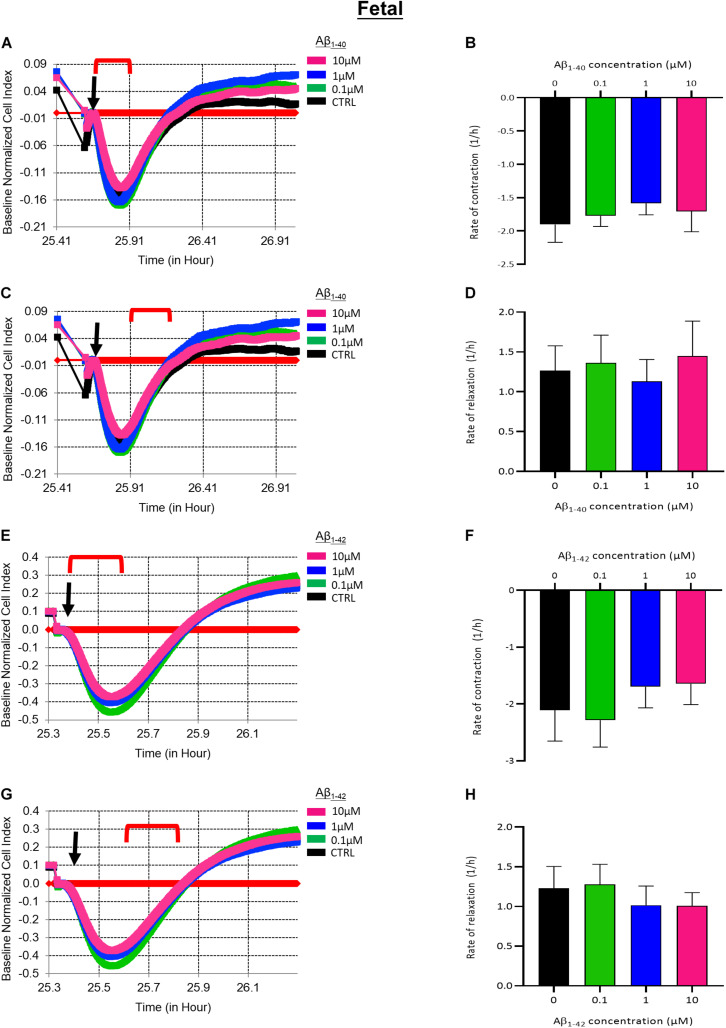
**(A)** Representative electrical impedance measurements of the contractile response of fetal human brain vascular pericytes (fHBVP) to EDN1 (100 nM) after 1 h incubation with Aβ_1__–__40_ (*n* = 1). **(B)** There was no significant difference between the rate of contraction of EDN1-treated cells following pre-exposure to Aβ_1__–__40_ (*n* = 7). **(C)** Representative electrical impedance measurements of the relaxation response of fHBVP following contraction (*n* = 1). **(D)** There was also no significant difference between the rate of subsequent relaxation EDN1-treated cells following pre-exposure to Aβ_1__–__40_ (*n* = 7). **(E)** Representative electrical impedance measurements of the contractile response of these fHBVP to EDN1 (100 nM) after 1 h treatment with Aβ_1__–__42_ (*n* = 1). **(F)** The differences in the rate of contraction between EDN1-treated cells following pre-exposure to Aβ_1__–__42_ were not statistically significant (*n* = 7). **(G)** Representative electrical impedance measurements of the relaxation response of fHBVP following contraction (*n* = 1). **(H)** There was also no significant difference in rate of subsequent relaxation (*n* = 7). The timeframe referred to in each graph is indicated by the red bracket. Arrows indicate the timepoint of EDN1 addition. The bars represent the mean values and SEM.

To investigate the acute effects of Aβ on EDN1-induced contraction of fHBVP we pre-incubated cells with Aβ_1__–__40_ or Aβ_1__–__42_ (10–0.1 μM) 1 h before the addition of EDN1 (100 nM). Neither contraction nor relaxation was significantly altered with exposure to Aβ_1__–__40_ or Aβ_1__–__42_ (Figures 6A–H).

**FIGURE 7:**
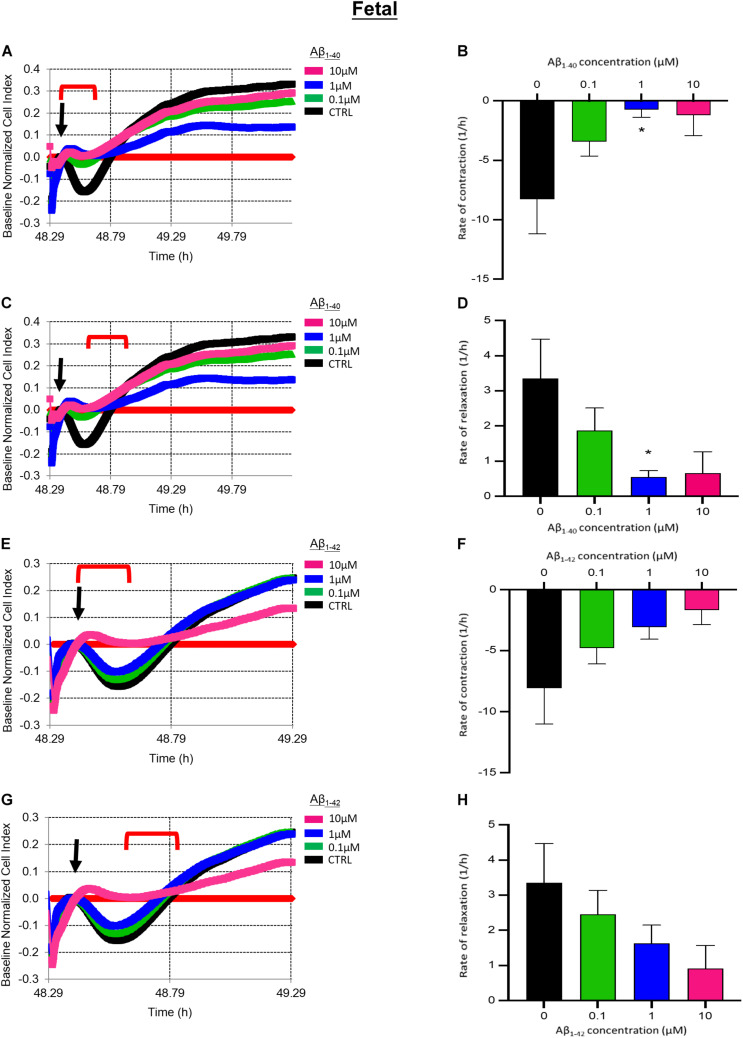
**(A)** Representative electrical impedance measurements of the contractile response of fetal human brain vascular pericytes (fHBVP) to EDN1 (100 nM) after 24 h incubation with Aβ_1__–__40_ (*n* = 1). **(B)** There was a significant difference between the rate of contraction of untreated cells and those exposed to 1 μm (*p* = 0.0249) Aβ_1__–__40_ (*n* = 9). **(C)** Representative electrical impedance measurements of the relaxation response of fHBVP following contraction (*n* = 1). **(D)** There was also a significant difference between the rate of subsequent relaxation in untreated of cells and those exposed to 1 μm (*p* = 0.0409) Aβ_1__–__40_ (*n* = 9). **(E)** Representative electrical impedance measurements of the contractile response of these fHBVP to EDN1 (100 nM) after 24 h treatment with Aβ_1__–__42_ (*n* = 1). **(F)** The differences in the rate of contraction between EDN1-treated cells following pre-exposure to Aβ_1__–__42_ were not statistically significant (*n* = 9). **(G)** Representative electrical impedance measurements of the relaxation response of fHBVP following contraction (*n* = 1). **(H)** There was also no significant difference in rate of subsequent relaxation (*n* = 9). The timeframe referred to in each graph is indicated by the red bracket. Arrows indicate the timepoint of EDN1 addition. The bars represent the mean values and SEM.

We next determined if longer exposure to Aβ peptides for 24 h impacted fHBVP contractility. Pre-treatment with 1 μM Aβ_1__–__40_ for 24 h significantly impaired EDN1-induced pericyte contraction (*p* = 0.0249) (Figures 7A,B). The subsequent increase in cell index (reflecting relaxation) was also significantly slower in cells treated with 1 μM Aβ_1__–__40_ (*p* = 0.0409) (Figures 7C,D). The contractile response to EDN1 tended also to be lower after pre-treatment with Aβ_1__–__42_ but the difference was not statistically significant (*n* = 6) (Figures 7E–H).

Exposure to Aβ_1__–__40_ and Aβ_1__–__42_ for 24 h at concentrations above 1 μM caused pericyte death, indicated by the incorporation of ethidium-1 dye. Pericyte death was minimal at Aβ_1__–__40_ and Aβ_1__–__42_ levels below 1 μM (Supplementary Figure 3).

It was not possible to assess the effects of prolonged exposure of aHBVP to EDN1 because of deteriorating viability and responsiveness of the adult cells after 24 h exposure to Aβ_1__–__40_ or Aβ_1__–__42_.

## Discussion

In this study, we have used electrical impedance assays (xCELLigence) to characterize the contractile and proliferative responses of fHBVP and aHBVP to EDN1. EDN1-induced contraction of fHBVP and aHBVP was mediated specifically by activation of EDNRA. The contractile responses were stronger in the fetal than adult cells, and the former proliferated after EDN1-induced contraction whereas the latter did not. Exposure to Aβ_1__–__40_ for 24 h significantly abrogated the contractile response to EDN1 in fHBVP, and subsequent relaxation. In the absence of EDN1, neither Aβ_1__–__40_ nor Aβ_1__–__42_ induced cell contraction.

Pericytes respond to vasoactive stimuli; ATP (Hørlyck et al., 2021) and EDN1 (Chakravarthy et al., 1992) induce pericyte contraction whereas adenosine and nitric oxide cause pericyte relaxation (Neuhaus et al., 2017). In human and rat cortical slices, noradrenaline constricts, and glutamate dilates capillaries in proximity to pericyte cell bodies (Peppiatt et al., 2006; Hall et al., 2014; Nortley et al., 2019). EDN1 was previously reported to cause pericyte contraction of bovine retinal pericytes (Chakravarthy et al., 1992), associated with the release of cytosolic Ca^2+^ and reorganization of actin filaments (Dehouck et al., 1997). More recently, EDN1 was shown to induce strong pericyte-mediated contraction reducing the lumina of capillaries in rat cortical slices – an effect mediated by the EDNRA receptor (Neuhaus et al., 2017; Nortley et al., 2019). Neuhaus et al. (2017) demonstrated the utility of xCELLigence to first measure contraction, relaxation and proliferation of human fetal brain-derived pericytes in response to EDN1 and various vascular stimuli. We have confirmed that EDN1 induces contraction of fHBVP, across a similar dose range to that previously reported (1 nM–1 μM), reflecting the normal concentration of EDN1 in human plasma (3.55 ± 1.78 ρg/ml) (Kostov et al., 2016). The contractile response was dose-dependent and was typical of a classical G-protein coupled response (Billington and Penn, 2003). We have extended the previous findings and demonstrated that both fHBVP and aHBVP are responsive to EDN1, and that the contraction is mediated through activation of the EDNRA receptor. Exposure to EDN1 alone does not cause pericyte toxicity, with longer exposure causing cell proliferation between 48 and 72 h (Neuhaus et al., 2017).

Fetal pericytes were more responsive to EDN1. The minimum concentration of EDN1 needed to elicit contraction in aHBVP was 0.1 μM–100 times greater than needed for contraction of fHBVP. aHBVP also took longer to relax following contraction, as demonstrated by the slower increase in cell index following contraction compared to that for fHBVP. Impaired relaxation of aHBVP could potentially affect neurovascular coupling and contribute to cerebral hypoperfusion in normal aging. Unlike fHBVP, which proliferated in response to EDN1 as reported previously (Neuhaus et al., 2017), aHBVP did not. Whether this reflects a genuine physiological difference between pericytes obtained from fetal and adult brains or is specific to this commercial source of pericytes (Cell-Systems), remains to be determined. Further studies to characterize the morphology and physiology of fetal and adult brain-derived pericytes and their anatomical subtypes should provide important insights into age- and disease-related pericyte dysfunction.

Soluble Aβ peptides, consisting of monomeric and oligomeric species cause a slow constriction of brain capillaries, mediated by pericyte contraction, reducing the diameter of the vessel lumen by up to 25% in human and rat cortical sections (Nortley et al., 2019). Transgenic rats treated with Aβ_1__–__40_ in the lateral ventricles showed an increase in EDNRA expression in the cerebral cortex, hippocampus, and brain stem (Briyal et al., 2011). We therefore wondered whether pre-exposure to Aβ had an impact on EDN1-induced contraction of fHBVP and aHBVP *in vitro*.

Short-term exposure had no effect. However, 24 h exposure of fHBVP to Aβ_1__–__40_, at concentrations reported in the human brain (∼6 nM) (Nortley et al., 2019), significantly altered the contractile response of pericytes to EDN1. Higher concentrations of Aβ (in the micromolar range) impaired the contractile responses of pericytes but this may have been partly because of cell toxicity. Previous studies found that exposure to 5–25 μM Aβ_1__–__40_ over 3–7 days caused degenerative changes (Verbeek et al., 1997; Sagare et al., 2013) whereas 1.4 μM for 3 h did not cause pericyte death in rat cortical slices (Nortley et al., 2019). We have not found evidence of pericyte degeneration at concentrations of Aβ_1__–__40__or_ Aβ_1__–__42_ below 1 μM over a 24 h period. Relaxation of fHBVP was also impaired after exposure to Aβ_1__–__40_. The deleterious impact of Aβ on pericyte responsiveness – slowing and reducing the contractile responses as well as subsequent relaxation – is likely to interfere with the rapid adaptation of capillary blood flow to changes in neuronal metabolic demand, and suggests a mechanism whereby elevated Aβ may contribute to neurovascular uncoupling and cerebral hypoperfusion in AD. It remains to be determined the extent to which the aggregation states of Aβ (monomeric, oligomeric, and fibrillar), influences pericyte contractile function and the impact of Aβ on pericyte responses to vasodilators such as NO, adenosine and PGE_2_, also merits further study.

Limitations of this study include the reliance on single sources and batches of human fetal and adult pericytes. Pericytes are likely to show heterogeneity of responsiveness *in vivo*, because of multiple genetic and epigenetic factors. Although the fetal pericytes have been used in numerous studies and express canonical pericyte receptors including PDGFRβ, NG2, and desmin (Neuhaus et al., 2017), we are still unsure of the precise CNS source of these cells and our observations need to be confirmed by study of pericyte function *in vivo*. Recent transcriptomic studies, such as Vanlandewijck et al. (2018) suggest that these cells may represent vascular fibroblast-like cells rather than pericytes (*personal communication*) especially after long term culture. This needs further analysis, although we would note that our studies were all on cells that had been passaged fewer than 8 times. We have used a reductionist approach to examine pericytes in isolation whereas *in vivo*, the actions of EDN1 may potentially be modulated locally by neurons, endothelial cells, astrocytes, and microglia. Lastly, we have relied on electrical impedance measurements as proxy indicators of contraction and proliferation. EDN1 was previously shown to mediate proliferation in a study that used both xCELLigence and traditional trypan-blue cell counting (Neuhaus et al., 2017), and we have used a BRDU assay to confirm pericyte proliferation in response to EDN1 (Supplementary Figure 2). Other methods, such as measuring the relative change in cell area by confocal microscopy (Hørlyck et al., 2021), are likely to be useful in future *in vivo* studies to validate our findings.

In conclusion, we have shown that fetal and adult brain-derived pericytes contract in response to activation of EDNRA by EDN1. Adult pericytes were less sensitive than fetal pericytes to EDN1 and remained contracted for longer. The contractile response of fetal pericytes was impaired after chronic exposure to Aβ. These differences may be relevant to disease pathogenesis and should be considered in the design of future studies.

## Data Availability Statement

The original contributions presented in the study are included in the article/Supplementary Material, further inquiries can be directed to the corresponding author.

## Author Contributions

EH performed all the experiments, analyzed and interpreted the data, and prepared the first draft of the manuscript. All authors contributed to design of the study, revised and edited subsequent drafts of the manuscript, contributed to the article, and approved the submitted version.

## Conflict of Interest

The authors declare that the research was conducted in the absence of any commercial or financial relationships that could be construed as a potential conflict of interest.

## Publisher’s Note

All claims expressed in this article are solely those of the authors and do not necessarily represent those of their affiliated organizations, or those of the publisher, the editors and the reviewers. Any product that may be evaluated in this article, or claim that may be made by its manufacturer, is not guaranteed or endorsed by the publisher.

## Supplementary Material

Supplementary Figure 1 **(A)** Graphical representation of cell contraction on impedance plates. Contraction reduces the cell surface area in contact with culture dish electrodes represented by a reduction in cell index. **(B)** Example impedance trace readout from the xCELLigence software. Image **(B)** taken and adapted from ACEA Biosciences Inc.

Supplementary Figure 2 Fetal human brain-derived vascular pericytes (fHBVPs) were seeded in a 96-well plate at 5,000 cells per well and left to settle overnight. Pericytes were incubated with purified recombinant EDN1 (diluted in serum free medium) for 72 h (*n* = 1). A BRDU incorporation assay (Abcam, United Kingdom) was used according to the manufacturer’s instructions to assess pericyte proliferation.

Supplementary Figure 3 **(A)** Fetal human brain-derived vascular pericytes (fHBVPs) and **(B)** adult human brain derived-vascular pericytes (aHBVP) were seeded in black clear bottomed 96-well plates at 5,000 cells per well and left to settle overnight. Pericytes were then incubated with recombinant Aβ peptides (rPeptide, diluted in 35% acetonitrile as a stock and diluted in serum-free medium) for 24 h. Cell toxicity was assessed using an ethidium dye, in the live/dead mammalian cell assay (Invitrogen, United Kingdom), according to the manufacturer’s guidelines (*n* = 2). The percentage of cells that had died was calculated with reference to the untreated control wells. The bars represent the mean values and SD.
