# Supplementary figures and images for: Pericyte Contractile Responses to Endothelin-1 and Aβ Peptides: Assessment by Electrical Impedance Assay
Source: Front Cell Neurosci. 2021 Aug 20;15:723953. doi: 10.3389/fncel.2021.723953 (PMC8417582; doi:10.3389/fncel.2021.723953)

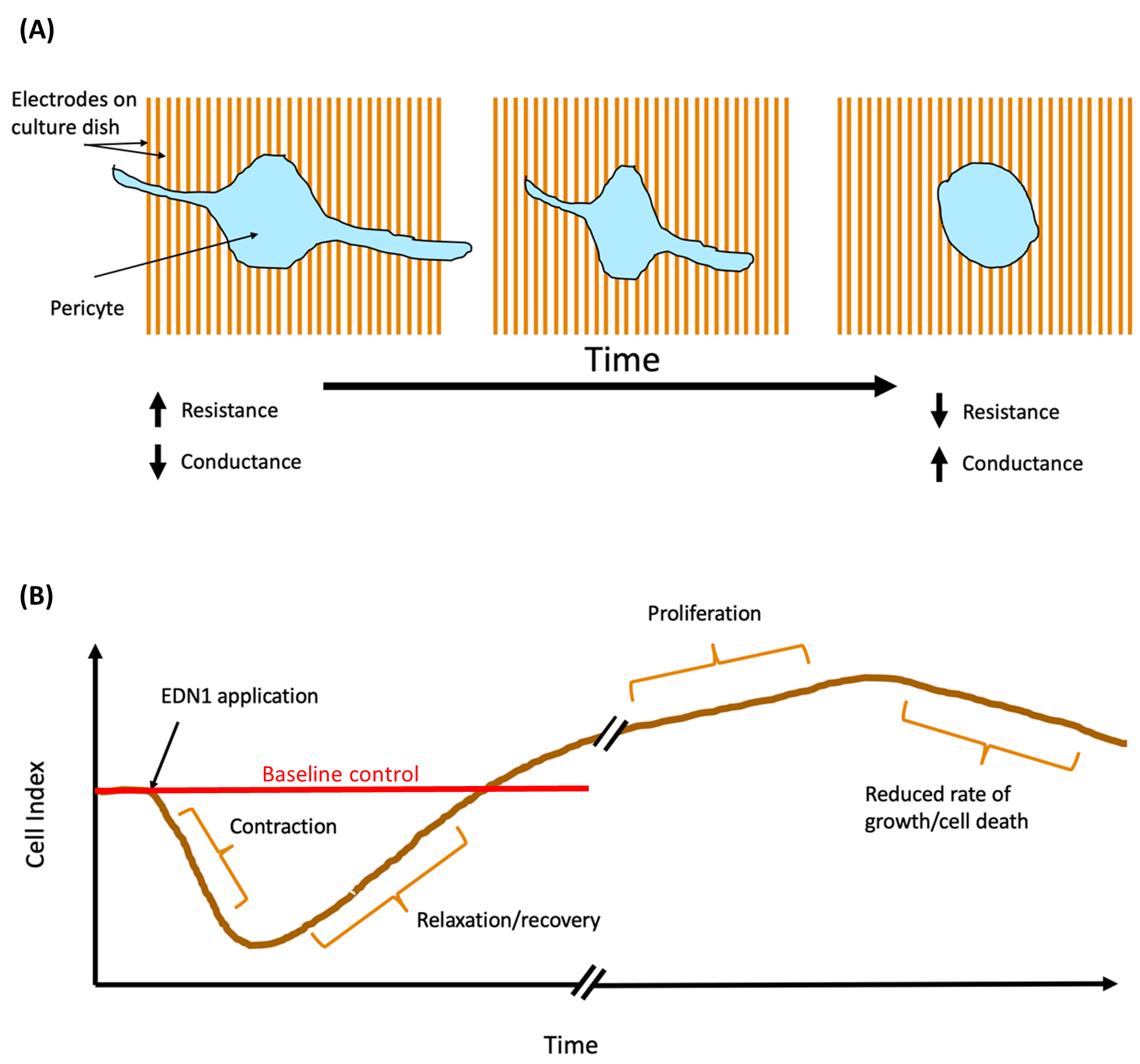

Supplement: Supplementary Figure 1 — (A) Graphical representation of cell contraction on impedance plates. Contraction reduces the cell surface area in contact with culture dish electrodes represented by a reduction in cell index. (B) Example impedance trace readout from the xCELLigence software. Image (B) taken and adapted from ACEA Biosciences Inc. [file Image_1.TIF]

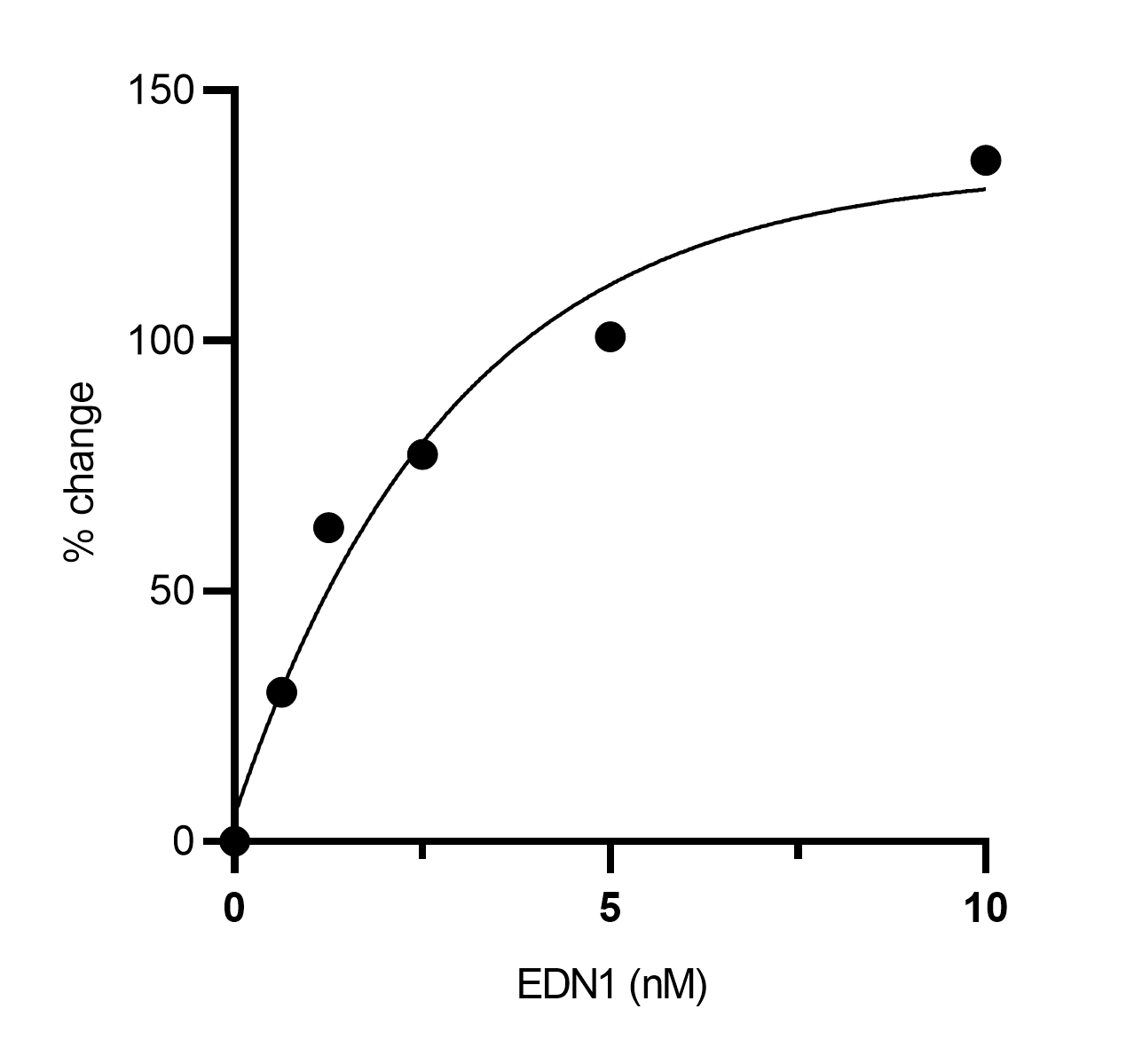

Supplement: Supplementary Figure 2 — Fetal human brain-derived vascular pericytes (fHBVPs) were seeded in a 96-well plate at 5,000 cells per well and left to settle overnight. Pericytes were incubated with purified recombinant EDN1 (diluted in serum free medium) for 72 h (n = 1). A BRDU incorporation assay (Abcam, United Kingdom) was used according to the manufacturer’s instructions to assess pericyte proliferation. [file Image_2.TIF]

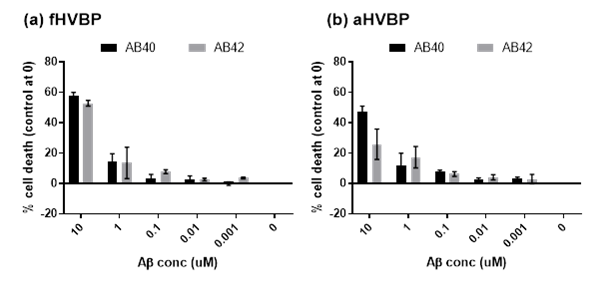

Supplement: Supplementary Figure 3 — (A) Fetal human brain-derived vascular pericytes (fHBVPs) and (B) adult human brain derived-vascular pericytes (aHBVP) were seeded in black clear bottomed 96-well plates at 5,000 cells per well and left to settle overnight. Pericytes were then incubated with recombinant Aβ peptides (rPeptide, diluted in 35% acetonitrile as a stock and diluted in serum-free medium) for 24 h. Cell toxicity was assessed using an ethidium dye, in the live/dead mammalian cell assay (Invitrogen, United Kingdom), according to the manufacturer’s guidelines (n = 2). The percentage of cells that had died was calculated with reference to the untreated control wells. The bars represent the mean values and SD. [file Image_3.TIF]
